# Intertubular morphometric and ultrastructural testes analyses in mdx mice

**DOI:** 10.1590/1984-3143-AR2021-0124

**Published:** 2022-10-24

**Authors:** Janine Karla França da Silva Braz, Vilessa Araújo Gomes, Verônica Andrade Siman, Sérgio Luís Pinto da Matta, Naianne Kelly Clebis, Moacir Franco de Oliveira, Antônio Chaves Assis, Danielle Barbosa Morais, Carlos Eduardo Bezerra de Moura

**Affiliations:** 1 Escola Multicampi de Ciências Médicas do RN, Universidade Federal do Rio Grande do Norte, Caicó, RN, Brasil; 2 Departamento de Ciências da Saúde, Universidade Federal de Campina Grande, Campina Grande, PB, Brasil; 3 Departamento de Biologia Geral, Universidade Federal de Viçosa, Viçosa, MG, Brasil; 4 Departamento de Morfologia, Universidade Federal do Rio Grande do Norte, Natal, RN, Brasil; 5 Departamento de Ciência Animal, Universidade Federal Rural do Semi-Árido, Mossoró, RN, Brasil; 6 Departamento de Cirurgia, Faculdade de Medicina Veterinária e Ciência Animal, Universidade de São Paulo, São Paulo, SP, Brasil

**Keywords:** seminiferous tubules, telocyte, testicles, myopathies, leydig cells

## Abstract

Duchenne Muscular Dystrophy (DMD) reproductive alterations and the influence of antioxidant treatments may aid in understanding morphometry testicular quantification. In this context, the aim of the present study was to characterize the intertubular compartment (ITC) morphometry of animal testes in mdx mice supplemented with ascorbic acid (AA). Sixteen mice were used, namely the C57BL/10 (non-dystrophic) and C57BL/10Mdx (dystrophic) lineages, distributed into the following groups: Control (C60), Dystrophic (D60), Control supplemented with AA (CS60), Dystrophic supplemented with AA (DS60). A total of 200 mg/kg of AA were administered to mice for 30 days. Subsequently, the testicles were collected, weighed, and fragmented. The obtained fragments were fixed in Karnovsky's solution (pH 7.2) and embedded in historesin for morphometric and transmission electron microscopy assessments. Leydig cells were hypertrophic in the D60 group, but was reverted by AA supplementation in the DS60 group. The DS60 group also exhibited increased intertubular volume compared to the CS60 group. The ultrastructural images identified multilamellar bodies in dystrophic animals (lipid storage) and telocyte cells (transport substances) in both control and dystrophic animals. Morphometric alterations were, therefore, noted in the intertubular compartment due to Duchenne muscular dystrophy (DMD), with AA administration capable of altering Leydig cells in this condition.

## Introduction

Duchenne Muscular Dystrophy (DMD) is the most common muscular dystrophy, comprising a lethal recessive genetic disease attached to the X chromosome that leads to progressive muscular degeneration due to the absence of the dystrophin protein. DMD affects 1 in every 3,500 males, and clinical signs usually develop in early childhood, where carriers present difficulties when running or climbing stairs and suffer frequent falls when walking (Kieny et al., 2013). Cardiac, respiratory, gastrointestinal, and orthopedic complications may also occur with dystrophy progression (Bushby et al., 2010). Animal models are often employed in the study of muscular dystrophy, with mdx mice the most adequate to assess myonecrosis and regeneration mechanisms in DMD, aiming at developing therapeutic strategies. mdx mice develop recessive muscular dystrophy associated with the X chromosome (locus Xp21, mutation in exon 23), and do not express the dystrophin protein (Almeida et al., 2016).

Some reports indicate human carriers of another type of dystrophy, Myotonic Muscular Dystrophy (MD1), who also present hypogonadism, affecting tubular and interstitial testicular function and leading to testosterone production alterations and erectile dysfunction (Peric et al., 2013). Free and total testosterone levels in serum have been determined in different dystrophinopathies, including DMD, revealing that 54% of carriers present low total testosterone levels, 39% present low free and total testosterone levels and 8% present low free testosterone levels and normal total testosterone levels (Al-Harbi et al., 2008).

There is evidence that high reactive oxygen species (ROS) levels may aggravate muscular dystrophies, initiating apoptosis in testicular germ cells (Rao and Shaha, 2000; Tidball and Wehling-Henricks, 2007). Thus, the use of antioxidants such as ascorbic acid (AA) may similarly affect the testicular parenchyma, also reducing muscular necrosis promoted by oxidative stress (Dorchies et al., 2006). In fact, according to Sönmez et al. (Sönmez et al., 2005) the use of AA significantly increases the testosterone levels and fertility rates of Wistar mice.

Testosterone synthesis is performed through Leydig cells, located in the interstitium (Fawcett et al., 1973; Setchell, 2004). These cells contains lipid droplets (LD) in their cytoplasm and can produce and secrete ﻿multilamellar bodies (MLBs) associated with LD (Haseeb et al., 2019). MLBs have been found near blood vessels or telocyte cells (Haseeb et al., 2019), the latter comprising a new cell found in the peritubular compartment. Although the function of this cell has not yet been elucidated, studies propose their participation in testes lipid synthesis (Sanches et al., 2021).

Cell quantification through the application of intertubular morphometry analyses may provide important data regarding gonadal activity and fertility (Antônio et al., 2002; Morais et al., 2014; Russell, 1990). However, no studies reporting the effects of AA in Leydig cells nor the morphometric quantification of such cells in DMD carriers are available to date.

In this context, this study furthers knowledge concerning new successful treatments for dystrophinopathies, ensuring increased life expectancy for carriers. This is evidenced when comparing the life expectancy of DMD carriers in the 1960's, of 14.4 years old to current data indicating carriers can live to be over 40 (Kieny et al., 2013; Saito et al., 2017). These factors can contribute to the increasing search for reproductive intervention alternatives through genetic counseling and innovative therapies by DMD carriers, such as CRISPR-Cas9 (Bengtsson et al., 2017; Dalton et al., 2015; Wallace et al., 2005). In this sense, this work aims to characterize the effect of an intertubular compartment treatment based on AA triggered by DMD.

## Methods

### Animals

Sixteen male mice were employed, eight belonging to the C57BL/10 strain (control group, non-dystrophic), originating from the Fiocruz/Rio de Janeiro bioterium, and eight belonging to the C57BL/10Mdx (dystrophic animals) from the University of São Paulo/ICB/USP. The project was approved by the CEUA/UFRN ethics committee (Protocol nº 064/2013-CEUA/UFRN).

The pubescent 60-day old mice mice were divided into four groups, namely the control group (C60), the dystrophic group (D60), the control group supplemented with AA (CS60), the dystrophic group supplemented with AA (DS60). The animals were maintained in the Anatomy Department animal house at ICB/USP in polyethylene boxes containing a water cooler and a feeder at a controlled temperature of about 22°C and under a 12-hour light/dark cycle.

### Supplementary AA diet

After weaning at 21 days old, the animals received commercial rodent feed (Nuvilab^®^, Nuvital, São Paulo, Brazil). At 30 days old, the animals from both AA-supplemented group (CS60 and DS60) received 200 mg/kg animal weight of AA diluted in water, prepared daily (Sigma-Aldrich, St. Louis, USA) to avoid oxidation, administered via gavage for 30 days. The AA concentration was calculated considering established literature protocols (Guido et al., 2010; Tonon et al., 2012).

### Euthanasia and sample collection

The animals were euthanized after the experimental period employing a hermetically sealed carbon dioxide chamber with a gas entrance in the upper portion of the chamber. Following euthanasia, the animals were weighed and their testicles collected, weighed, transversally sectioned, and fixated by immersion in Karnovsky’s solution for 24 h for testicular morphometry light microscopy and ultrastructural electronic transmission microscopy (ETM) analyses (Karnovsky, 1965; Morais et al., 2014).

### Histological processing

The testis were measured (length and width) to calculate the retraction factor (5%) to correct morphometric values prior to the histological processing. The testicular fragments destined for the morphometry analyses were dehydrated in an increasing ethanol series (70 to 100%) using glycol-methacrylate (Historesin^®^ LeicaMycrosistems, Heidelberg, Germany). Histological semi-sectioned sections (3 µm thick) were obtained using a Leica RM2255rotary microtome (Leica Microsystems, Heidelberg, Germany), with 40 µm intervals between cuts, and blades dyed with toluidine blue/1% sodium borate. The preparations were analyzed under a Motic BA410 microscope (Motic, CausewayBay, Hong Kong) and micro-photographed using a digital 5.0 MP Moticam camera (Motic Instruments Inc, Richmond, Canada) employing the Motic Images Plus 2.0 OML software (Motic, Xiamen, China).

The samples were fixed in Karnovsky’s solution for 24 h and then washed in phosphate buffer at 0.1 M (pH 7.4). After fixing, the fragments destined for the ultrastructural cellular analyses were washed three times in a phosphate buffer at 0.1 M pH 7.4 for ten minutes each and post-fixated with 2% osmium tetroxide buffered in sodium phosphate 0.1M, pH 7.4 for two hours. The fragments were washed three more times in a buffered solution for ten minutes each, immersed overnight in uranyl acetate at 3%, washed in a buffered phosphate and dehydrated in an increasing ethanol series (50 to 100%) for ten minutes each, followed by immersion in propylene oxide for ten minutes to ensure complete tissue dehydration. Subsequently, the materials were blocked in 502 araldite resin (Polysciences Inc, California, USA). Semi-thin cuts were obtained employing an ultra-microtome and stained with a 1% toluidine blue aqueous solution to identify adequate areas to obtain ultra-thin cuts. Ultrathin sections (70 nm thick) were then obtained with a diamond razor and placed in 200 mesh copper screens for subsequent contrast with uranyl acetate saturated at 2% from seven to ten minutes, followed by lead citrate at 0.5% for the same amount of time. The material was finally analyzed under a Jeol^®^ 100 CX II TEM (Tokyo, Japan).

### Testicular stereology

The weight of the tunica albuginea was estimated from testicular volume density (Vv), by counting 266 points projected over 10 images obtained from the histology preparations of each animal under a 10x objective lens (Vv = Number of points counted throughout the tunica albuginea/Total of counted points x 100). The absolute volume of the albuginea was the result of the product of testicular density and volume by testicular volumes, considering a testicular density of of about 1 (Costa et al., 2011; Johnson et al., 1981; Sprando et al., 1998).

The volumetric ratios between the seminiferous tubules and the intertubule were estimated by counting 266 points projected over 10 images for each animal applying the following equation: (Number of points counted throughout the tubule or intertubule/Total of counted points x 100). The percentage of each element of the intertubular compartment was quantified from the projection of 1,000 points over the intertubule of each animal, quantifying the coincident points over the Leydig cells nuclei and cytoplasm, as well as blood vessels, lymphatic space, and connective tissue. The percentages of each of these components in the intertubule and testicles were estimated from the following equations, respectively: Number of points counted over the element x 100/ 1000 and % of intertubule x % of the element in the intertubule/100, while their volumes were estimated as % of the element in the testicle x weight of the testicular parenchyma/100 (Morais et al., 2014). All counts were performed using the Image-Pro Plus® software (Media Cybernetics Inc., Rochville, USA).

Leydig cell nuclear diameters were measured when circular contouring perinuclear chromatin, and evident nucleoli were observed, by quantifying 30 nuclei per animal. Leydig cell nuclear volume (NV) and cytoplasmic volume (CV) per animal were expressed as µm^3^, as follows: NV=4/3 πR^3^, with R comprising the nuclear radius and CV = % of cytoplasm x NV/% nucleus. The cellular volume was obtained by summing up NV + CV.

The total number of Leydig cells was obtained by dividing the total volume of these cells per testicular parenchyma (µm^3^) by the volume of each Leydig cell (µm^3^). The number of cells per gram of testicle was obtained by dividing the TLC by the total gonadal weight. The Leydigosomatic index, which quantifies the investment in Leydig cells with regard to body mass, was obtained as ILS = total volume of Leydig cells per testicular parenchyma/ PC x 100, where PC = body weight.

### Statistical analyses

Quantitative testicular parameters were expressed as the means ± standard deviations and submitted to a variance analysis (ANOVA) and the Kruskall-Wallis’s multiple comparison test followed by Mann-Whitney’s test with Bonferroni’s correction, using the PAST^®^ software version 2.17 (Hammer et al., 2001). The employed significance level was P≤0.05. Comparisons between C60-DS60 and CS60-D60 were not considered.

## Results

Dystrophic animals supplemented with AA exhibited increased gonad (0.17 ± 0.03 g) and intertubule (0.04 ± 0.01 μl) weight and volume compared to CS60 animals (0.13 ± 0.02 g and 0.02 ± 0.001 μl, respectively). The dystrophy condition increased the lymphatic parenchyma space in the D60 group (9.8 ± 1.3 μl) compared to both the C60 (4.3 ± 1.2 μl) and DS60 (10.3 ± 2.5 μl) group compared to CS60 (5.6 ± 0.8 μl) (Table 1)

**Table 1:** Biometric and morphometric testicular intertubular compartment data in mice with and without Duchenne Muscular Dystrophy supplemented or not with ascorbic acid.

**Variables**	**C60**	**CS60**	**D60**	**DS60**
Body weight (g)	23.37±2.62	21.77±1.16	28.95±1.16	27.93±1.16
Gonadal weight (g)	0.13±0.01^Aa^	0.13±0.02^Ca^	0.14±0.01^Ac^	0.17±0.03^Dc^
Seminiferous tubule (%)	74.25 ± 6.00	80.12 ± 2.24	72.58 ± 2.80	75.67 ± 4.42
Intertubule (%)	25.75 ± 6.00	19.88 ± 2.24	27.42 ± 2.80	24.33 ± 4.42
Seminiferous tubule (mL)	0.090±0.02	0.10±0.02	0.10±0.01	0.12±0.01
Intertubule (µL)	32.4±8.8^Aa^	24.2±1.4^Ca^	38.1±7.2^Ac^	40.3±16.7^Dc^
Intertubule (%)				
Leydig cells	16.13±5.21	10.23±5.55	9.93±4.18	8.80±5.69
Blood vessels	63.08±14.55	61.70±8.96	61.00±10.90	61.28±1.24
Lymphatic space	14.58±6.71	23.15±3.23	26.43±6.76	26.65±4.65
Connective tissue	6.23±3.43	4.93±1.15	2.65±1.02	3.28±1.30
Testicular parenchyma (%)				
Leydig cells	4.05±1.61	2.09±1.39	2.65±1.02	2.33±1.91
Bloodv essels	16.69±6.76	12.20±1.85	16.82±4.29	14.89±2.99
Lymphatic space	3.56±1.56	4.61±0.98	7.20±1.83	6.32±0.42
Connective tissue	1.46±0.54	0.97±0.23	0.74±0.32	0.78±0.28
Testicular parenchyma (µL)				
Leydig cells	4.9±1.2	2.4±1.2	3.6±0.9	4.1±3.5
Bloodv essels	21.3±9.9	15.0±2.8	23.8±8.4	24.7±9.9
Lymphatic space	4.3±1.2^Aa^	5.6±0.8^Ca^	9.8±1.3^Bc^	10.3±2.5^Dc^
Connective tissue	1.8±0.5	1.2±0.2	1.0±0.4	1.3±0.6

Means presenting different pairs of capital letters on the same line (A-B; C-D) indicate comparisons between control (C60 x D60 and supplemented (CS60 x DS60) groups, respectively. Means presenting different pairs of lower-case letters on the same line (a-b; c-d).

All groups comprised 60 day-old mice. C60: control group; CS60: control group supplemented with ascorbic acid, D60: Dystrophic group; DS60: Dystrophic group supplemented with ascorbic acid. Means presenting different pairs of capital letters on the same line (A-B; C-D) indicate comparisons between control (C60 x D60 and supplemented (CS60 x DS60) groups, respectively. Means presenting different pairs of lower-case letters on the same line (a-b; c-d) indicate comparisons between control (C60 x CS60) and dystrophic (D60 x DS60) groups, respectively. Data are expressed as means ± standard deviations of the means (p≤0.05).

The dystrophy condition in pubescent animals (D60) significantly increased nuclear (466.03 ± 59.32 µm^3^) and cytoplasmic (1921.62 ± 637.12µm^3^) Leydig cell volumes compared to C60 animals (70.66 ± 10.35 µm^3^ and 231.01 ± 34.10 µm^3^, respectively). Consequently, individual Leydig cell volume increased in pubescent dystrophic mice (D60) compared to the C60 group (2387.66 ± 665.54 µm^3^ *versus* 301.68 ±40.33 µm^3^). Supplementation with AA in the dystrophic group (DS60) significantly reduced nuclear (62.08 ± 17.43µm^3^), cytoplasmic (224.90 ± 117.06µm^3^) and individual (286.98 ± 131.80µm^3^) Leydig cell volumes compared to the D60 group (Table 2).

**Table 2:** Leydig cell morphometry and testicular intertubular compartment leydigosomatic rates in mice with and without Duchenne Muscular Dystrophy supplemented or not with ascorbic acid.

**Variables**	**C60**	**CS60**	**D60**	**DS60**
Nuclear diameter (µm)	5.12±0.25^Aa^	4.79±0.37^Ca^	9.61±0.39^Bc^	4.88±0.50^Cd^
Nuclear percentage (%)	23.49±2.47	20.48±5.23	20.41±4.54	22.97±4.92
Nuclear volume (μm^3^)	70.66±10.35^Aa^	58.22±13.51^Ca^	466.03±59.32^Bc^	62.08±17.43^Cd^
Cytoplasmatic percentage (%)	161.25±52.07	102.25±55.46	99.25±41.77	88.00±56.90
Cytoplasmatic volume (μm^3^)	231.01±34.10^Aa^	233.71± 59.25^Ca^	1921.62± 637.12^Bc^	224.90± 117.06^Cd^
Leydig cell volume (μm^3^)	301.68±40.33^Aa^	291.93±61.51^Ca^	2387.66±665.54^Bc^	286.98±131.80^Cd^
Leydig cells/testis (10^7^)	1.65±0.39^Aa^	0.91±0.63^Ca^	0.15±0.02^Bc^	1.49±1.61^Cd^
Leydig cells/gram of testis (10^7^)	13.41±4.65^Aa^	8.08±7.02^Ca^	1.11±0.26^Bc^	8.30±7.21^Cd^
Leydigosomatic index (%)	0.022± 0.009^Aa^	0.011 ±0.005^Ca^	0.0123± 0.0030^Bc^	0.0152±0.0158^Cc^

Means presenting different pairs of capital letters on the same line (A-B; C-D) indicate comparisons between control (C60 x D60 and supplemented (CS60 x DS60) groups, respectively. Means presenting different pairs of lower-case letters on the same line (a-b; c-d) indicate comparisons between control (C60 x CS60) and dystrophic (D60 x DS60) groups, respectively.

All groups comprised 60 day-old mice. C60: control group; CS60: control group supplemented with ascorbic acid, D60: Dystrophic group; DS60: Dystrophic group supplemented with ascorbic acid. Means presenting different pairs of capital letters on the same line (A-B; C-D) indicate comparisons between control (C60 x D60 and supplemented (CS60 x DS60) groups, respectively. Means presenting different pairs of lower-case letters on the same line (a-b; c-d) indicate comparisons between control (C60 x CS60) and dystrophic (D60 x DS60) groups, respectively. Data are expressed as means ± standard deviations of the means (p≤0.05).

The dystrophy condition increased nuclear Leydig cell diameters, with AA supplemented dystrophic mice (DS60) exhibiting significantly reduced nuclear Leydig cell diameters (4.88 ± 0.50 µm) compared to the D60 group (9.61± 0.39 µm) (Table 2). The dystrophy condition significantly reduced the Leydigosomatic index in D60 animals compared to the C60 group (0.0123 ± 0.0030% *versus* 0.022 ± 0.009%). This was also observed for the number of Leydig cells per gram of testicle in the D60 group compared to the C60 group (1.11 ± 0.26 x 10^7^ *versus* 13.41 ± 4.65 x 10^7^) (Table 2).

**Figure 1:**
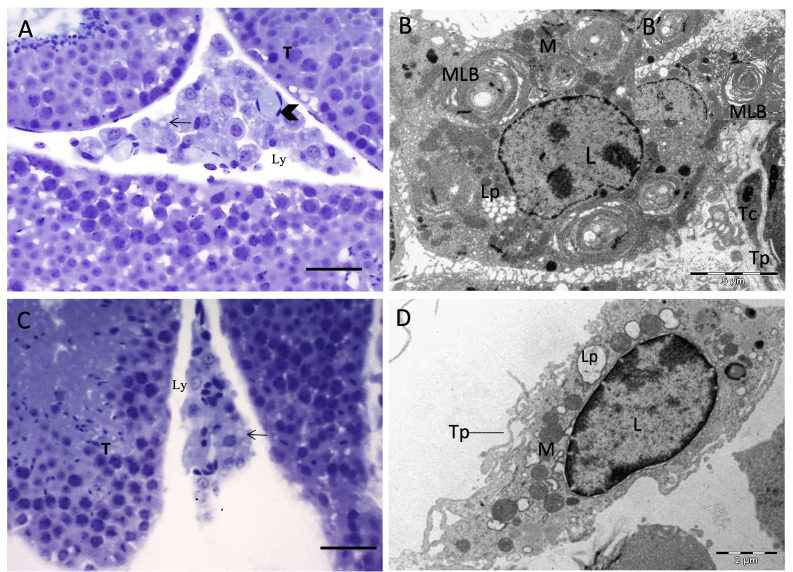
Leydig cells in mice with Duchenne Muscular Dystrophy from the experimental groups. (A) Small lipid vesicle agglomerates in C60. B: C60 Leydig cell with rounded nucleus and lipid vesicle agglomerates; (B) Multilamellar bodies in Leydig Cells; (C) D60 reduced and spaced lipid vesicles; (D) D60 rounded nucleus in Leydig cell presenting rounded mitochondria and lipid vesicles and telopodes. (T) Seminiferous tubule; (L) Leydig cell Nuclei; (Ly) lymphatic space; (Arrow) (Lp) lipid vesicles; (arrow tip) Blood vessel; (MLB) Multilamellar body; (M) mitochondria; (Tc) Telocyte; (Tp) Telopode. Toluidine Blue Color (light microscopy) - Bar = 20 µm.

**Figure 2:**
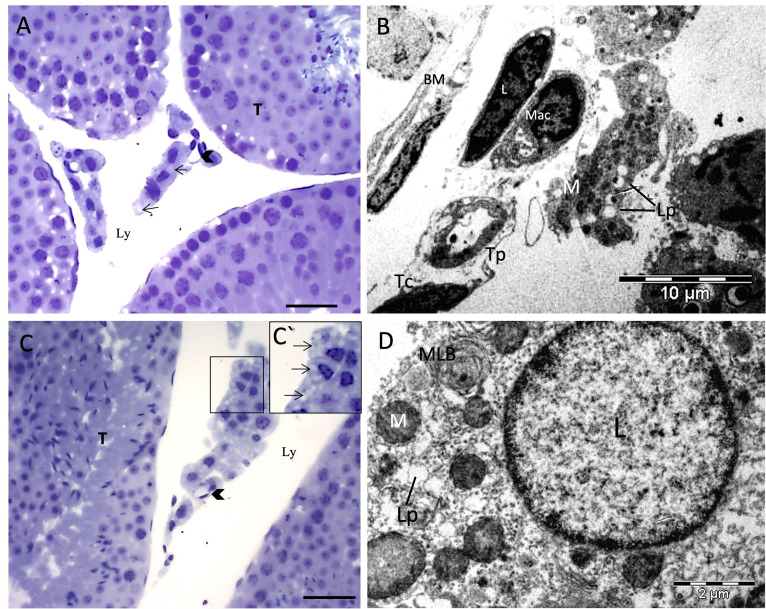
Leydig cells in mice from the experimental groups supplemented with ascorbic acid. (A) CS60, discrete lipid vesicles; (B) CS60, Leydig cells with elongated nucleus, several mitochondria and lipid vesicles dispersed in the cytoplasm with telocytes; (C) DS60, discrete lipid vesicles in Leydig cytoplasm. C`: DS60, magnified lipid droplets; (D) DS60, Leydig cells with irregular nucleus, spaced lipid vesicles, and multilamellar bodies. (T) Seminiferous tubule; (BM) basal membrane; (Mac) macrophage; (M) mitochondria; (L) Leydig cells; (Ly) lymphatic space; (Arrow) (Lp) lipid vesicles; (Tip of the arrow) Blood vessel; (MLB) Multilamellar body; (Tp) Telopode; (Tc) Telocyte. Toluidine Blue Color (light microscopy) - Bar = 20 µm.

The light microscopy analysis evidenced small lipid droplets in the C60 group (Figure 1A), forming agglomerates, absent in the D60 group (Figure 1C). Leydig cell cytoplasms in both the CS60 group and supplemented DS60 dystrophic group exhibited discrete lipid droplets (Figure 2A and Figure 2C).

Ultrastructural analyses were employed to assess Leydig cell morphology, indicating elongated nuclei and irregular aspects in the D60 group, while the C60, CS60 and DS60 groups presented elongated to rounded nuclei. Circular mitochondria were observed in the Leydig cells of all groups. Lipid drop agglomerates were confirmed in Leydig cell cytoplasm compared to the dystrophic group, and the TEM analyses revealed the presence of multilamellar bodies in both the control group (C60) and Dystrophy (DS60) groups (Figure 1B and Figure 2D), with lipid droplets noted in all groups (Figure 1B and D; Figure 2B and Figure 2D). Telocytes cells in both the control and dystrophic group were observed in the peritubular region and near the Leydig cells (Figure 1B and Figure 2B). Telocytes are small, long and thin cells containing a heterochromatin nucleus and peripherical mitochondria in with a discrete nucleolus, also exhibiting extensions termed podomers (Figure 2B).

## Discussion

Mammal testicles can be functionally categorized into two compartments, namely the tubular or spermatogenic compartment and the intertubular or androgenic compartment. Most mammals present Leydig cells as an important functional constituent of the intertubular compartment, which secrete steroids and essential pheromones that act in the sexual behavior of males and in spermatogenesis development (França and Russell, 1998). An increase in amounts of Leydig cells in the testicular parenchyma during the pre-pubescent phase is widespread in mammals, regressing until puberty (Setchell, 2004). the findings reported herein indicate that DMD may comprise a significant driver in elevating the nuclear and cytoplasmic volume of Leydig cells and, consequently, their individual volume in puberty, characterizing a hypertrophy. This alteration in mice exposed to formaldehyde fumes has shown to lead to decreased testosterone rates resulting from functional Leydig cell failure (Razi et al., 2013). However, AA supplementation in dystrophic mice may revert this condition, probably due to increased glutathione levels, which in turn decrease ROS levels responsible for promoting testicular oxidative stress, similarly to what has been reported for the combined treatment of AA and a chemotherapy in mice presenting tumors (Longchar and Prasad, 2015). The percentage of intertubular and tubular compartments may vary according to species and factors responsible for differences in spermatic production efficiency (França and Russell, 1998; Russell, 1990). The intertubule analyzed herein in mice is morphologically similar to the descriptions noted for other mammals in both the control and dystrophic groups, composed of Leydig cells, blood vessels, connective tissue and lymphatic space (França and Russell, 1998). Our findings confirm increases in blood vessel density, which may be associated to active lymphagiogenesis in mdx mice, a process previously reported for mdx mice (Santos et al., 2013). However, AA treatment is not able to reduce this mechanism, as DMD can promote inflammatory processes in several organs (Seixas et al., 1997).

The ultrastructural analyses revealed that Leydig cell nuclei are morphologically rounded and contain heterochromatin in both the control and 60-day-old supplemented dystrophic groups. However, dystrophic animals presented irregular nuclei, despite Hooker’s description of Leydig cells as normal when ellipsoid or polygonal (Hooker, 1970). According to previous reports in mice treated with herbicides, this nuclear Leydig cell pleomorphism may reduce plasmatic testosterone levels and lead to other structural changes (Victor-Costa et al., 2010).

The Leydig cells assessed herein contained lipid droplets, a formed by cholesterol esters, which comprise a substrate for testosterone biosynthesis catalyzed by enzymes located in the smooth endoplasmic reticulum and mitochondria membranes (Mori and Christensen, 1980). Furthermore, telocytes were evidenced in both control and dystrophic group testes. Telocytes are a new type of classic interstitial cells first described in the early 21^st^ century (Hinescu and Popescu, 2005) present in several organs, including the human testicle (Marini et al., 2018) and mdx mice testes (Gomes et al., 2021). Although their function has not yet been elucidated, some hypotheses indicate that these cells may be involved in the synthesis of testis lipids in the genital system (Sanches et al., 2021), is due to the communication of cytoplasmic processes through telopods (Tp), with multilamellar bodies (lipid storage) located in the Leydig cell cytoplasm (Haseeb et al., 2019). Furthermore, the presence of testicular telocytes in dystrophic mice seems to imply in spermatogenesis mechanisms, potentially aiding in seminiferous tubuletestosterone uptake (Gomes et al., 2021). Thus, it is possible that these cells may play a role in the testis of dystrophic mice, although further investigations on this specific topic are required.

Most domestic mammals present an average of 20-60 million Leydig cells per gram of testicle (França and Russell, 1998). This parameter can be used to monitor testosterone levels, as a positive correlation between Leydig cells per gram of testicle tissue and plasmatic testosterone levels is noted in mammals, for example, normal rabbits (Castro et al., 2002). Thus, as the dystrophy condition considerably reduces the number of Leydig cells per gram of testicle, it may also reduce plasmatic testosterone levels during the pubescent period. Plasmatic testosterone concentrations re an important parameter that should be further evaluated to confirm potential correlations in this regard.

Decreased gonadal weight and increased Leydig cell volume in dystrophic animals have been implicated in the proportional body mass increase of Leydig cells, as well as in the Leydigosomatic index compared to control animals. This allows for comparisons between different -ized species, as Golden Retriever dogs are commonly used as a model for DMD (Martins-Júnior et al., 2015). In addition, this rate varies according to animal age in normal dogs with no defined breed, increasing until puberty when greater spermatic production takes place, decreasing in the post-pubescent period (Mascarenhas et al., 2006). This behavior comprising greater body mass Leydig cells investment in dystrophic mice (D60) is certainly another compensatory action to ensure greater hormonal support for spermatogenesis.

## Conclusion

DMD may affect the volumetric component ratios of the intertubular compartment, especially concerning Leydig cell number, morphology and ultrastructure. Supplementation with AA may reverse the accentuated Leydig cell hypertrophy observed in dystrophic pubescent mice.
